# Investigating depression, anxiety, perceived stress and resilience in fathers faced with their spouse’s abortion in Iran: a longitudinal study

**DOI:** 10.1186/s12888-024-05887-w

**Published:** 2024-07-08

**Authors:** Seyede Zahra Jafari, Mahboubeh Hajifoghaha, Sara Azima, Parvin Ghaem Maghami, Zahra Yazdan Panahi

**Affiliations:** 1 Department of Midwifery, Student Research Center, School of Nursing and Midwifery, Shiraz University of Medical Sciences, Shiraz, Iran; 2 Department of Midwifery and Reproductive Health, School of Nursing and Midwifery affiliated to Shiraz, University of Medical Sciences, Shiraz, Iran; 3 Department of Midwifery, School of Nursing and Midwifery, Shiraz University of Medical Sciences, Shiraz, Iran; 4 Department of Biostatistics, Medical School, Shiraz University of Medical Sciences, Shiraz, Iran; 5 Maternal-Fetal Medicine Research Center, Department of Midwifery, School of Nursing and Midwifery, Shiraz University of Medical Sciences, Shiraz, Iran

**Keywords:** Abortion, Depression, Anxiety, Perceived stress, Resilience, Fathers

## Abstract

**Background:**

Abortion is a stressful event that can often affect the mental health of both parents. It seems that resilient people can adapt to stressful situations. The mental health of fathers plays an important role in improving the mental health of the family, but few studies have been conducted in this regard. Therefore, this study aimed to investigate depression, anxiety, perceived stress and resilience of fathers faced with their spouse’s abortion.

**Methods:**

This longitudinal study was conducted on 125 spouses of women hospitalized in the post-partum department of Shiraz hospitals in 2023. Data collection tools included questionnaires of demographic and fertility characteristics, hospital depression and anxiety (HADS), Cohen’s perceived stress, and Connor’s resilience. The data were analyzed through Spss24 software using Friedman’s tests and post hoc tests, Adjusted Bonferroni, Kruskal-Wallis and Mann-Whitney tests.

**Results:**

The mean age of the fathers was 35.02 *±* 6.22. The scores of the father’s anxiety, depression, and perceived stress from 24 h to 12 weeks after abortion were decreased significantly. However, their resilience score increased significantly. Also, there was a significant relationship between the fathers’ age, education, job, duration of marriage, type of abortion, number and history of abortion, unwanted pregnancy, number of children and economic status with the mean score of anxiety, depression, perceived stress, and resilience in fathers over time.

**Conclusion:**

This research pointed out the effect of abortion on depression, anxiety, and perceived stress in fathers; also, resilience as a coping factor could affect these disorders and improve the fathers’ mental health. Therefore, screening and managing mental disorders in them are important to improve family health.

## Introduction

Fertility is highly valued in most cultures and the desire to have a child is one of the most basic human motivations in forming a family. If the attempt to conceive and have a child fails, it can become a destructive emotional experience [1]. Abortion is one of the causes of pregnancy failure that is not diagnosed in most cases [2]. Abortion occurs in 15–25% of pregnancies that are clinically identified before the 20th week of pregnancy [2, 3] The National Center for Health Statistics, CDC^1^ and WHO^2^ defined abortion as the termination of pregnancy before the 20th week or the birth of a fetus weighing less than 500 g. More than 80% of spontaneous abortions occur in the first 12 weeks of pregnancy; also, as to its occurrence between the first 5–20 weeks of pregnancy, its rate is variable and about 10–20% has been stated [4]. Abortion can often involve both parents with mental disorders [5]. Research on the impact of abortion on fathers began in 1960; then, numerous quantitative and qualitative studies in different societies helped to better understand the issue [6]. When faced with abortion, men experience the same emotions as women, although with less intensity and for a shorter period [7, 8]. The psychological reaction caused by abortion in fathers has been reported from immediately to one year after the abortion [8]. According to studies, in different types of early pregnancy loss in their wives, men experience different emotions such as depression and sadness [6]. Major depressive disorder is a mental illness that causes social, economic problems and emotional burden leading to disruption in social or occupational functioning. Therefore, it disrupts a person’s daily life. It is also a common, recurrent and costly disorder, causing frequent disabilities [9]. Symptoms of major depressive disorder according to DSM-V^3^ include symptoms of depressed mood (sadness, emptiness, or hopelessness) or loss of interest or pleasure in daily activities, along with social impairment, occupational, and educational functioning [10]. Studies show that in fathers faced with abortion, initial depression and anxiety will be high in the first month and then decrease over a year. However, this reduction was not uniform; for example, some studies indicated an increase in the aforementioned disorders in the third month compared to the first month [11, 12]. Anxiety is a pervasive, unpleasant, and vague disturbance that is often accompanied by symptoms of the autonomic nervous system such as headache, sweating, palpitations, feeling of tightness in the chest, and slight stomach discomfort [13]. The experience of moderate to severe anxiety can be caused by parents immediately after abortion [11, 14]. According to Hans Selye, the Austrian physician and the father of stress, stress refers to the body’s physical-chemical, mental, and emotional reactions to sensitive and dangerous events and situations [15]. Abortion can be interpreted as a psychological factor causing stress. because it constitutes an exhausting or even threatening situation, which is interpreted as stressful for most people [16, 17].

Few studies have been conducted to assess the long-term outcome of abortion. Therefore, there is very little knowledge about those adaptive factors, such as resilience, that shape the trajectory of early pregnancy adaptation [18].

Resilience is an ability to adapt positively in stressful situations and includes thoughts, behaviors, or actions that every person can learn. Of course, resilience not only is stability in the face of injuries or threatening conditions, or a passive state in facing dangerous conditions, but is considered an active company in the environment around you. In addition, resilience helps biological-psychological balance in dangerous and stressful situations. It is also one of the appropriate ways to deal with an adverse event and leads to higher self-efficacy, better coping, and less perception of stress [19–21]. The current study was conducted to investigate the three-month trend of depression, anxiety, perceived stress, and resilience of fathers faced with abortion in 3 hospital centers affiliated with Shiraz University of Medical Sciences, Iran. Considering the few studies on the effect of abortion on the mental health of fathers and its effect on the health of the whole family, this study was conducted aiming to improve the process of providing midwifery services.

## Materials and methods

This is a longitudinal study to investigate the three-month trend of depression, anxiety, perceived stress, and resilience in 125 fathers who had their wives’ experienced abortion in the last 24 h, using the available sampling method. It was done in the postpartum departments of three hospital centers, Shiraz, Iran in 2023.

The sample size was determined 114, considering α = 0.05 (first type error) β = 0.20 (second type error), 95% confidence factor and 90% power using G* Power3 software, based on the default effect size of Classification of the good area (Cohen’s effect size 0.3 and f = 0.15 effect size) and correlation 0.4 between the measurement times, Given the possibility of a drop of 10%. 125 subjects in total were determined to be enrolled.

$$\text{n}={\rho }\frac{{({\text{z}}_{1-\frac{{\alpha }}{2}}+{\text{z}}_{1-{\beta }})}^{2}}{{\left(d\right)}^{2}}$$

### Data collection tools

Data collection tools were presented to the participants in the first 24 h in person, then 4 weeks, and 12 weeks after abortion by national social media.

1. Demographic and fertility questionnaire.

2. HADS^4^ was designed by Sigmond and Snaith (1983); it has 14 questions used to measure mood changes, especially anxiety and depression states. In this scale, there are seven questions (odd questions) related to anxiety symptoms and seven questions (even questions) related to depression symptoms. This questionnaire is scored based on a four-point scale. The highest score is 3 and the lowest score; each person will have a score between zero and twenty-one in each area. A score higher than 10 is considered as probable mood disorder [22]. This tool has been validated and reliable in Iran by Montazeri and colleagues in 2003, and Cronbach’s alpha coefficient was 0.87 [23].

3. The perceived stress questionnaire was introduced by Cohen et al. in 1983. This 14-item scale measures perceived stress during the past month. This questionnaire is scored based on a five-point Likert scale, with never = 0, low = 1, medium = 2, high = 3and very high = 4 [24, 25]. The minimum and maximum scores that can be obtained on this scale are 0 and 56, respectively, and the cut-off point is 21.8; also, a score higher than that indicates high perceived stress [26]. Cohen and colleagues have reported the test-retest reliability of the scale as 0.80 [24]. The reliability of the Persian version was calculated by Bastani et al. in 1987 with the internal consistency method, and Cronbach’s alpha coefficient was 0.74 [27].

4. Connor and Davidson’s resilience questionnaire (adult resilience) prepared in 2003 by reviewing the research conducted in 1979–1991 in the field of resilience, contains 25 items and is scored using a five-point Likert scale of totally incorrect = 1, Rarely true = 2, mostly true = 4, Sometimes true = 3, and always true = 5. The minimum score of the questionnaire is 25 and the maximum 125. A score between 25 and 41 indicates low resilience 41 to 83 moderate resilience and a score above 83 high resilience [28]. Mohammadi in Iran obtained its validity and reliability based on Cronbach’s alpha of 0.93 and confirmed its validity [29].

### Data collection

The study data we collected during a period of 6 months from April to September 2023 in three selected hospital centers, The inclusion criteria were being Iranian, being 18 years and above, having minimum reading and writing literacy, living with one’s spouse, having had an abortion in the last 24 h, not having history of depression (based on self-report), not having a history of infertility or couples undergoing infertility treatment, not having experienced problems affecting mental health, not taking antidepressants and narcotics in the last 6 months, and completing the informed consent form: also, failure to answer more than 10% of the questionnaire questions as the exclusion criterion.

### Statistical analysis method

The data were analyzed using SPSS version 24 software using descriptive and analytical indicators. The normality of the distribution of depression, anxiety, perceived stress, and resilience scores was evaluated using the Kolmogorov-Smirnov test. At the level of descriptive statistics, statistical indicators include frequency (percentage) and average (standard deviation), to describe demographic characteristics; also, to compare the average scores of anxiety, depression, perceived stress, and resilience over time, we used Friedman’s tests and post hoc tests. Adjusted Bonferroni, Kruskal-Wallis and Mann-Whitney tests were used to check the relationship between variables and demographic characteristics.

## Results

125 eligible fathers filled out the questionnaires.

Demographic characteristics: Most of the fathers were under 35 years old (56.8%), had a diploma or higher education (73.6%), were self-employed (72.8%), had a medium and good economic status (85.6%) and mostly less than 5 years had passed since their marriage. 51.2% of fathers had no children. 43.2% of men had a history of abortion in their wives, and 52% of men experienced spontaneous abortion in their wives. Table 1.

**Table 1:** Socio-demographic profile of the participants *N* = 125

Variable		Frequency (%)
**Father age**		
• 18–23	**Age- Mean + SD, Range** **(35.02 + 6.22, 18–47)**	4 (3.2)
• 24–29		4 (3.2)
• 30–35		**63(50.4)**
• 36–41		40(32)
• 42–47		14(11.2)
**Duration of marriage**		
• Less than 5 years		**55(44)**
• 5–10 years		28(22.4)
• 10–15 years		19(15.2)
• More than 15 years		23(18.4)
**Education**		
• elementary		3(2.4)
• Secondary school		26(20.8)
• High school		4(3.2)
• diploma		**55(44)**
• university		37(29.6)
**Fathers job**		
• Free job		**91(72.8)**
• Employee		34(27.2)
**Economic Situation**		
• poor		14(11.2)
• medium		**60(48)**
• good		47(37.6)
• Very good		4(3.2)
**Number of children**		
• 0		**64(51.2)**
• 1		34(27.2)
• ≥ 2		27(21.6)
**Abortion history**		
• yeas		54(43.2)
• no		**71(56.8)**
**Abortion number**		
• 0		**71(56.8)**
• 1		50(40)
• 2		4(3.2)
**Abortion type**		
• Spontaneous		**65(52)**
• induce		52(41.6)
• recurrent		8(6.4)
**Unwanted pregnancy**		
• Yeas		43(34.4)
• No		**82(65.6)**

The fathers’ mean scores of anxiety, depression, perceived stress, and resilience when faced with their spouses’ abortion were determined and compared at 24 h, 4 weeks, and 12 weeks after abortion; based on the results of the Friedman test, there was a significant decrease in the scores of anxiety, depression, and perceived stress. A significant increase in the resilience score was observed three times compared to each other. According to the results of Bonferroni corrected post-hoc test, this significant difference was caused by the difference between the mean anxiety 12 weeks and 24 h after the abortion (-1.5 ± 2.8) (*P*-value = 0.010) and the mean anxiety12 weeks and 4 weeks after abortion (-1.1 ± 1.7) (*P*-value < 0.001). However, there was no significant difference between the mean anxiety 24 h and 4 weeks after abortion (-0.4 ± 3.17) (*P*-value = 0.087). As to depression, a significant difference was caused by the difference between the mean depression 12 weeks and 24 h after abortion (-1.8 ± 2.51) (*P*-value < 0.001) and that 12 weeks and 4 weeks after abortion (-1.19 ± 2.00) (*P*-value < 0.001); also, there was a significant difference between the mean depression 24 h and 4 weeks after abortion (-0.6 ± 2.15) (*P*-value = 0.018). There was a significant difference between the mean stress 12 weeks after abortion with that 24 h after abortion (-3.94 ± 3.37) (*P*-value < 0.001) and the mean stress 12 weeks and 4 weeks after abortion (-1.84 ± 3.12) (*P*-value < 0.001). In addition, there was a significant difference between the mean stress 24 h and 4 weeks after abortion (-2.09 ± 3.30) (*P*-value = 0.001). A significant difference was caused by the difference between the mean resilience 12 weeks after abortion with that 24 h´s after abortion (6.22 ± 4.87) (*P*-value < 0.001) and the mean resilience 12 weeks and 4 weeks after abortion (2.75 ± 4.44) (*P*-value < 0.001). In addition, there was a significant difference between the mean resilience 24 h and 4 weeks after abortion (3.47 ± 5.38) (*P*-value < 0.001). Table 2.

**Table 2:** Comparison of mean scores of anxiety, depression, perceived stress, and resilience of fathers in 24 h, 4 weeks, and 12 weeks after experiencing abortion

Time	24 h (1) Mean ± SD	4 week (2) Mean ± SD	12 week (3) Mean ± SD	*P*-value (Between)	Times ^b^	Mean Difference (SE)	*P*-value ^a^ ^(Within)^
**Group**							
anxiety Mean (SD)	3.66 ± 3.52	3.26 ± 1.84	2.15 ± 1.87	< 0.001	1–2 2–3 1–3	-0.40 (3.17) -1.10 (1.70) -1.50 (2.80)	0.087 < 0.001 0.010
Depression Mean (SD)	5.82 ± 1.84	5.21 ± 1.89	4.02 ± 2.16	< 0.001	1–2 2–3 1–3	− 0.60 (2.15) -1.19 (2.27) -1.80 (2.51)	0.018 < 0.001 < 0.001
Perceived stress (SE)	23.77 ± 4.25	21.68 ± 3.05	19.83 ± 3.20	< 0.001	1–2 2–3 1–3	-2.09 (3.30) -1.84 (3.12) -3.94 (3.37)	0.001 < 0.001 < 0.001
Resilience Mean (SD)	85.52 ± 6.29	89.0 ± 3.99	91.75 ± 3.38	< 0.001	1–2 2–3 1–3	3.47 (5.38) 2.75 (4.44) 6.22 (4.87)	< 0.001 < 0.001 < 0.001

^a^ Bonferroni post hoc test

^b^ Pairwise Comparison

There was a relationship between socio-demographic characteristics and the wife’s fertility like the fathers’age, education, fathers’ job, duration of marriage, abortion type, abortion-number, abortion history, unwanted pregnancy, number of children and economic-status with the scores of anxiety, depression, perceived stress, and resilience of fathers who had encountered with their spouses’ abortion (*P* > 0.050). Table 3.

**Table 3:** Correlation between the changes in the scores of 12 weeks compared to 24 h after abortion variables of anxiety, depression, perceived stress, and resilience with demographic and fertility information

Variable	Category	anxiety	depression	Perceived stress	resilience
		Standard deviation ± mean	Standard deviation ± mean	Standard deviation ± mean	Standard deviation ± mean
Fathers age	18–23	3 ± 0.00	-1 ± 0.00	-3 ± 0.00	5 ± 0.00
	24–29	-4 ± 0.00	-1 ± 0.00	-5 ± 0.00	10 ± 0.00
	30–35	-1.77 ± 2.8	-1.71 ± 2.6	-3.8 ± 3.84	6.36 ± 5.14
	36–41	-0.47 ± 1.8	-1.65 ± 2.3	-3.32 ± 2.5	5 ± 5.16
	42–47	-3.85 ± 3.3	-3.07 ± 2.8	-6 ± 3.28	8.35 ± 2.30
*P*-value†		**0.001**	0.580	**0.046**	0.061
duration of marriage	Less than 5 years	-1 ± 2.52	-1.6 ± 2.35	-4.72 ± 2.6	6.1 ± 3.58
	5–10 years	-1.21 ± 2	-1.92 ± 2.5	-2.32 ± 3.6	6.85 ± 4.51
	10–15 years	-2.68 ± 3.8	-1.57 ± 2.3	-4.15 ± 3.3	3.73 ± 5.99
	More than 15 years	-2.13 ± 3.2	-2.3 ± 2.97	-3.86 ± 4.0	7.78 ± 6.26
*P*-value†		0.460	0.799	**0.042**	0.162
education	elementary	-2 ± 0.00	-2 ± 0.00	-8 ± 0.00	10 ± 0.00
	Secondary school	-3.30 ± 3.6	-3.5 ± 2.64	-4.38 ± 3.3	6.53 ± 3.15
	High school	-4 ± 0.00	-3 ± 0.00	2 ± 0.00	11 ± 0.00
	diploma	-1.49 ± 2.5	2.34 ± 1.85	-4.65 ± 2.6	5.74 ± 4.51
	university	0.02 ± 1.86	-0.77 ± 2.0	-2.89 ± 3.68	5.89 ± 6.32
*P*-value†		**0.001**	**0.001**	**0.001**	0.073
Fathers job	Free job	-2.03 ± 3.01	-2.01 ± 3.4	-3.5 ± 3.65	6.29 ± 4.87
	Employee	-0.11 ± 1.71	-1.23 ± 2.5	-5 ± 2.1	6.02 ± 4.87
*P*-value†		**0.001**	0.093	**0.016**	0.863
economic situation	poor	-2.42 ± 3.58	-2.85 ± 3	-5.42 ± 3.75	4.64 ± 4.12
	medium	-2.2 ± 2.26	-1.75 ± 2.3	-2.98 ± 3.2	6 ± 4.72
	good	-0.7 ± 2.9	-1.61 ± 2.6	-4.8 ± 3.28	7 ± 5.37
	Very good	3 ± 0.00	-1 ± 0.00	-3 ± 0.00	5 ± 0.00
*P*-value†		**0.001**	0.730	**0.014**	0.391
Number of children	0	-0.81 ± 2.39	-1.25 ± 2.3	-4.87 ± 3.4	5.75 ± 3.77
	1	-1.47 ± 1.95	-2.32 ± 2.1	-3.11 ± 3.0	5.61 ± 4.49
	≥ 2	-3.22 ± 3.95	-2.44 ± 3	-2.77 ± 3.0	8.11 ± 6.93
*P*-value†		**0.031**	**0.047**	**0.004**	0.050
Abortion history	yeas	-1.4 ± 2.90	-1.16 ± 1.9	-3.88 ± 3.2	4.64 ± 5.15
	No	-1.5 ± 2.82	-2.28 ± 2.7	-3.98 ± 3.4	7.42 ± 4.3
*P*-value†		0.392	**0.015**	0.566	0.007
Abortion number	0	-1.59 ± 2.82	− 2.28 ± 2.78	-3 ± 3.48	7.42 ± 4.3
	1	-1.6 ± 2.93	-1.1 ± 2.02	-3.4 ± 2.84	5.02 ± 5.18
	2	1 ± 0.00	-2 ± 0.00	-10 ± 0.00	0.00 ± 0.00
*P*-value†		0.062	**0.034**	**0.003**	0.003
Abortion type	Spontaneous	-1.86 ± 2.83	-2.69 ± 2.42	-4.35 ± 2.67	7.01 ± 4.84
	induce	-1.3 ± 2.98	-0.73 ± 2.4	-3.26 ± 3.73	5.73 ± 4.91
	recurrent	0.000 ± 1.06	-1.5 ± 0.53	-5 ± 5.34	3 ± 3.2
*P*-value†		0.115	**0.001**	0.067	0.104
Unwanted pregnancy	yeas	-3.27 ± 3.0	-3.2 ± 2.39	-3.88 ± 3.1	7.39 ± 4.56
	No	-0.58 ± 2.2	-1.06 ± 2.25	-3.97 ± 3.5	5.6 ± 4.93
*P*-value†		**0.001**	**0.001**	0.948	0.003

†: Kruskal-wallis test

Mean score of anxiety, depression, perceived stress, and resilience of fathers 24 h, 4 weeks, and 12 weeks after experiencing abortion was different.

**Fig. 1:**
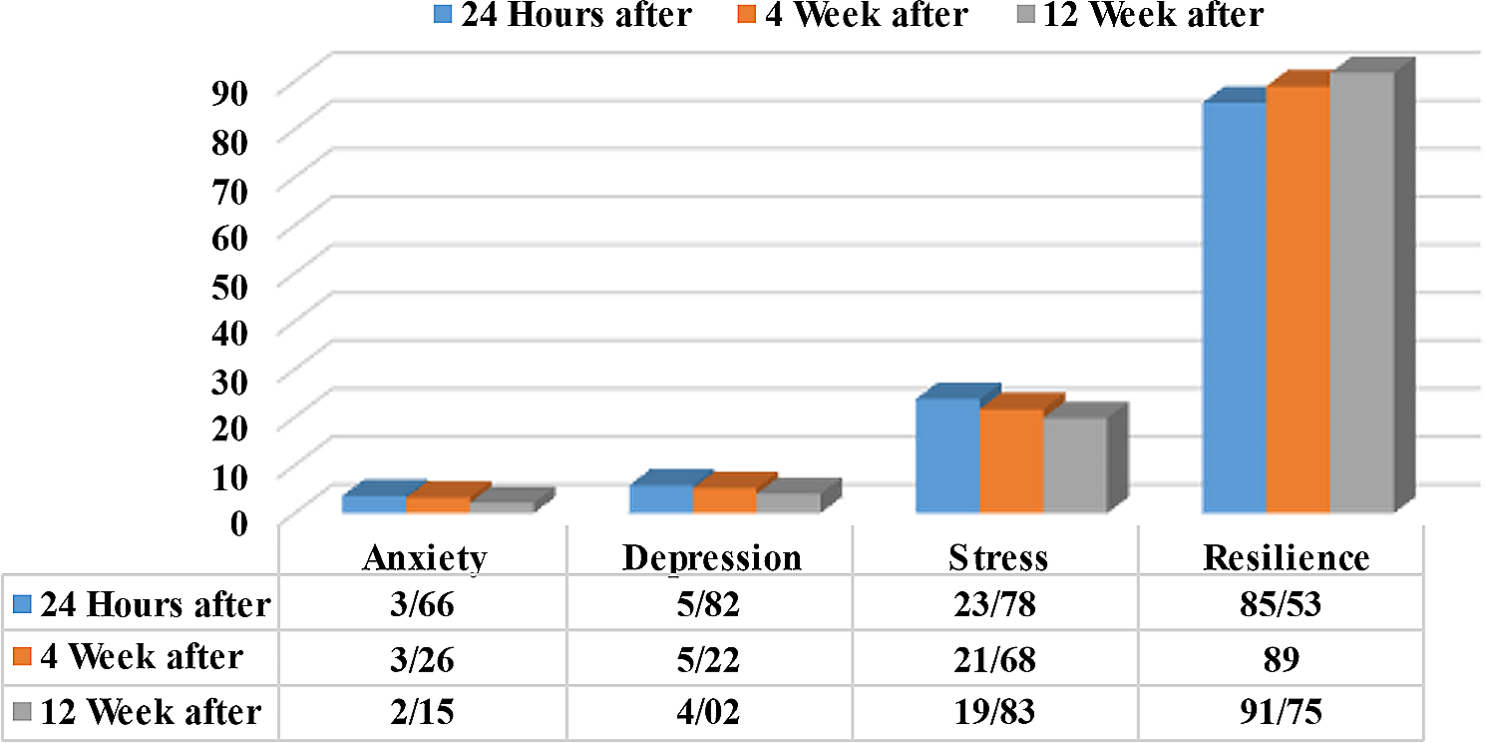
Mean score of anxiety, depression, perceived stress and resilience of fathers in 24 h, 4 weeks, and 12 weeks after experiencing abortion

According to the results a significant decrease in the fathers’ mean scores of anxiety (from 3.66 ± 3.52 to 2.15 ± 1.87), depression (from 5.82 ± 1.84 to 4.02 ± 2.16), and perceived stress (from 23.77 ± 4.25 to 19.83 ± 3.20), and a significant increase in the resilience mean score (from 85.52 ± 6.29 to 91.75 ± 3.38) was observed. Figure 1.

## Discussion

The present longitudinal study was conducted to investigate the three-month trend of depression, anxiety, perceived stress, and resilience of fathers whose wives had experienced abortion in Iran. The results obtained indicate a significant relationship between some demographic characteristics of the fathers like fathers age, education, fathers-job, duration of marriage, abortion type, number of abortion, abortion history, unwanted pregnancy, number of children, economic situation and the mental disorders under investigation. Another result of the study showed that the mean scores of the father’s anxiety, depression, and perceived stress from 24 h to 12 weeks after abortion were decreased significantly, and their resilience score increased significantly.

A cross-sectional study of Korn Ramp, which was conducted on 151 couples about 2–7 years after the termination of pregnancy due to abnormality, showed that higher education, good support from the sexual partner, lower gestational age, and incompatibility in life were among the factors involved in the development of mental disorders after abortion [30]. In a cross-sectional study by Kaplan et al., which was conducted on 35 patients and their spouses in Turkey, it was shown that the mean age of the participants was 33.74 ± 6.85 versus 35.02 *±* 6.22 in our study; 45.7% of them versus 29.6% in our study had a university education. 40% of them versus 11.2% in our study had a low income, and 11.4% of their subjects versus 21.6% in our study had more than two children. Also, the level of depression caused after curettage in their study was influenced by the job, social and private life, and physical health of mothers after induced abortion [31]. Although it did not examine the relationship between the fathers’ depression after induced abortion, it investigated some demographic factors examined in our study, and the relationship of them to the development of depression in fathers and mothers faced with abortion was discussed; due to smaller sample size and the difference in societies, different percentages of demographic factors have been obtained in the two studies.

Contrary to our study, Cumming’s prospective study was conducted in Scotland on 133 men and 273 women and showed that none of the demographic and fertility factors examined affected depression and anxiety scores over one year [32]. Anna Marie’s analytical study showed that among the risk factors affecting the resilience of mothers faced with induced abortion are the level of education, socio-economic conditions, mental and health status, history of mental disorders in parents, family conflicts and stressful events [33]. Although in this study the relationship between resilience and socio-demographic variables after facing abortion in fathers and over time has not been studied, the relationship between characteristics such as education level and socioeconomic conditions with mothers’ resilience after induced abortion has been pointed out. Contrary to Cumming’s prospective study, which was conducted in 3 early pregnancy assessment centers in Scotland on 273 women and 133 men at 1, 6 and 13 months after abortion, a change in psychometric scores was observed over more than one year after abortion [32] The longer time of Cumming’s study and the different sample sizes and cultural-social differences of the studied communities can play a significant role in creating differences in the results of the two studies.

Farren’s study followed 192 couples in London over 9 months after miscarriage or ectopic pregnancy. 7% of men met the criteria for post-traumatic stress syndrome in the first month, 8% in the third month, and 4% in the ninth month. Also, 6% had anxiety in the first month, 9% in the third month, 6% in the ninth month, 2% depression was seen in the first month, 5% in the third month and 1% in the ninth month [11].

In Farren’s study, fathers faced with abortion three months after abortion were faced with a higher level of anxiety, depression, and stress than one month later. This difference to our study, may be caused by difference in cultural, social, and economic issues, sample size, sampling method, and the use of different tools to measure stress (Cohen’s Perceived Stress Questionnaire in our study versus Post Traumatic Stress Questionnaire in Farren’s study) [11]. Contrary to our study, a prospective study by Delgado et al. was conducted in Spain on 29 mothers and 17 fathers after hospital discharge. All of them were over 18 years old. According to their results, there was no difference in the initial symptoms of depression and subsequent depression one month later in the couples. This difference may be caused by the difference in culture, sample size, and use of Edinburgh Questionnaire versus HADS Questionnaire to measure depression in parents after abortion [34].

A longitudinal study by Helena Volgsten on 64 Swedish couples faced with abortion showed that symptoms such as depression decreased four months after abortion compared to one week later [35]. Although the time frame of Helena Volgsten’s study is not the same as ours, both studies point to the decreasing process of depression of fathers faced with abortion over time. A review study by Lewis was conducted in Canada, and the highest prevalence of post-abortion depression in the first month was between 5 and 17% which decreased to 7% in the third month. This study showed that although women’s depression symptoms may have decreased at first, men’s depression symptoms may not be resolved as easily after abortion and may even remain stable for 1 year [12]. An integrated systematic review was conducted in Belgium by Lamon et al. They found that some studies showed an increased risk of depression and stress in fathers who had experienced pregnancy loss. However, the results of many studies did not show a significant difference, and, in the same line with our study, the symptoms also decreased over time [36]. Similar to our study, abortion was associated with depression, anxiety and high perceived stress in fathers; this is because of the low mean score of resilience as a coping responses at the first, and increase in the mean score of resilience during the time, as an ability to adapt to a traumatic experience and calamity or stressful event in life [37], can lead to the reduction of mental disorders after abortion. Couples’ resilience is actually a process that supports positive adaptation in the face of stressful life situations [38]. So far, no study has been conducted on the resilience over time in fathers faced with abortion, but our study showed that their resilience increased during the time. Anna Marie’s study also showed that stressful events in life like abortion, could affect the resilience of women faced with abortion [37]. A systematic review and meta-analytical study by Ghanei-Gheshlagh in Iran showed that resilience can maintain and improve mental health by providing cognitive, behavioral, and emotional responses in stressful situations [39]. Therefore, this study has also point to the importance of the role of resilience as a coping factor.

It is important to know and understand that there is a relationship between some socio-demographic characteristics and fertility characteristics of the spouse with anxiety, depression, perceived stress, and resilience of fathers because it can identify and eliminate the underlying factors of disorders. Also, paying attention to the fact that disorders such as depression, anxiety and perceived stress and the resilience of fathers faced with abortion change over time can play a significant role in determining the right time to provide the necessary care services and improve the mental health of fathers faced with abortion. Therefore, given this important point it is suggested that providers of pregnancy care should, consider the timely identification of possible mental disorders in fathers faced with abortion in the process of providing post-abortion care services and to carry out interventions. It is possible to improve the mental health of fathers as quickly as possible and ultimately improve the mental health of the family. Finally, the results of this study and other research show that attention should be paid to the prenatal care services for fathers after abortion.

This study is the first research in Shiraz, Iran that has determined the three-month trend of depression, anxiety, perceived stress, and resilience of fathers faced with abortion. The strength of this research can be the indication of the importance of the fathers’ participation, despite their neglect in the prenatal care program. But the secrecy of some abortion cases, the lack of consent and non-cooperation of some fathers, as well as the fact that some of them were not present when their wives were discharged from the hospital after the abortion, were among the limitations of the research.

## Conclusion

The findings of this research showed that the fathers’ mean score of depression, anxiety, and perceived stress during the period of three months decreased and, that of resilience increased. Given the high score of mental disorders after abortion during the first 24 h, it is recommended that healthcare professionals should identify fathers at risk and use interventional strategies to increase their resilience. It is suggested that this research should be used as a base for interventional studies and identification of predicting factors of depression, anxiety, perceived stress, and resilience. In addition, further studies with a larger sample size are also suggested.

## Data Availability

The datasets analyzed during the current study are not publicly available due to the confidentiality of participants’ data and the difficulty of organizing the raw data to be suitable for publication; however, they are available from the corresponding author on reasonable request.

## Footnotes

1 The Center for Disease Prevention and Control.

2 The World Health Organization.

3 DSM-V: Diagnostic and Statistical Manual of Mental Disorders.

4 The Hospital Anxiety and Depression Questionnaire.

## Abbreviations

CDC: Center for Disease Prevention and Control
HADS: Hospital Anxiety and Depression Questionnaire
